# SMYD2 Regulates Vascular Smooth Muscle Cell Phenotypic Switching and Intimal Hyperplasia via Interaction with Myocardin

**DOI:** 10.21203/rs.3.rs-2721176/v1

**Published:** 2023-04-13

**Authors:** Yu Zhou, Shaligram Sharma, Xiaonan Sun, Xiaoqing Guan, Yuning Hou, Zhe Yang, Hang Shi, Ming-Hui Zou, Ping Song, Jiliang Zhou, Shenming Wang, Zuojun Hu, Chunying Li

**Affiliations:** 1 Center for Molecular and Translational Medicine, Institute for Biomedical Sciences, Georgia State University, Atlanta, GA, USA.; 2 Division of Vascular Surgery, National-Local Joint Engineering Laboratory of Vascular Disease Treatment, Engineering and Technology Center for Diagnosis and Treatment of Vascular Diseases, Guangdong Engineering Laboratory of Diagnosis and Treatment of Vascular Disease, The First Affiliated Hospital, Sun Yat-Sen University, Guangzhou, China.; 3 Department of Biochemistry, Microbiology, and Immunology, Wayne State University School of Medicine, Detroit, MI, USA.; 4 Center for Obesity Reversal, Department of Biology, Georgia State University, Atlanta, GA, USA.; 5 Department of Pharmacology and Toxicology, Medical College of Georgia, Augusta University, Augusta, GA, USA.

**Keywords:** vascular smooth muscle cell, phenotypic switching, intimal hyperplasia, SMYD2, histone methylation, myocardin

## Abstract

The SET and MYND domain-containing protein 2 (SMYD2) is a histone lysine methyltransferase that has been reported to regulate carcinogenesis and inflammation. However, its role in vascular smooth muscle cell (VSMC) homeostasis and vascular diseases has not been determined. Here, we investigated the role of SMYD2 in VSMC phenotypic modulation and vascular intimal hyperplasia and elucidated the underlying mechanism. We observed that SMYD2 expression was downregulated in injured carotid arteries in mice and phenotypically modulated VSMCs *in vitro.* Using a SMC-specific *Smyd2* knockout mouse model, we found that Smyd2 ablation in VSMCs exacerbates neointima formation after vascular injury *in vivo.* Conversely, Smyd2 overexpression inhibits VSMC proliferation and migration *in vitro* and attenuates arterial narrowing in injured vessels in mice. Smyd2 downregulation promotes VSMC phenotypic switching accompanied with enhanced proliferation and migration. Mechanistically, genome-wide transcriptome analysis and loss/gain-of-function studies revealed that SMYD2 up-regulates VSMC contractile gene expression and suppresses VSMC proliferation and migration, in part, by promoting expression and transactivation of the master transcription cofactor myocardin. In addition, myocardin directly interacts with SMYD2, thereby facilitating SMYD2 recruitment to the CArG regions of SMC contractile gene promoters and leading to an open chromatin status around SMC contractile gene promoters via SMYD2-mediated H3K4 methylation. Hence, we conclude that SMYD2 is a novel regulator of VSMC contractile phenotype and intimal hyperplasia via a myocardin-dependent epigenetic regulatory mechanism and may be a potential therapeutic target for occlusive vascular diseases.

## Introduction

Vascular smooth muscle cells (VSMCs) are major cellular component of blood vessels, providing structural and functional support to maintain vascular homeostasis.^1^ Many vascular diseases, such as in-stent restenosis, vein bypass graft failure, and aneurysm, are largely dependent on VSMC phenotypic switch from a differentiated contractile state to a proliferative synthetic phenotype in response to endogenous and exogenous cues. During VSMC phenotypic switch, smooth muscle cell (SMC)-specific gene expression decreases while cell proliferation and migration increase, leading to vascular intimal hyperplasia.^2^ Therefore, elucidating the mechanisms underlying VSMC phenotypic switch is important to understand the pathology of proliferative vascular diseases and develop therapeutic approaches.^1,3^ Among various mechanisms that contribute to transcriptional regulation of VSMC marker genes, myocardin-serum response factor (SRF)-CArG [CC(A/T)_6_GG] axis is the best characterized regulatory circuit in VSMC differentiation.^4-6^ However, studies have showed that coordinate action of SRF and myocardin is not sufficient to control the expression of SMC marker genes, and the regulation of chromatin conformation and transcription factor accessibility by histone modifications is also crucial.^7-9^ Epigenetic modifications and chromatin remodeling are key processes in cell differentiation and control of cell-specific marker expression due to acquisition of a unique epigenetic signature.^10^ Among epigenetic modifications, histone methylation is an important determinant of distinct cell phenotypes.^11^ The central role for epigenetic complexes in the determination of SMC lineage fate has yet to be fully characterized.^3,12,13^ However, the importance of histone methylation in these processes has been recently recognized. ^7,8,14,15^

SET and MYND domain-containing protein 2 (SMYD2) belongs to SMYD family of lysine methyltransferases.^16,17^ SMYDs are a special class of protein methyltransferases that mediate lysine methylation of histones and non-histone proteins and are involved in transcriptional regulation, cell differentiation and proliferation, cancer progression, and muscle development.^18-20^ SMYD2 was originally reported to methylate histone 3 at lysine 36 (H3K36) and lysine 4 (H3K4),^16,21^ and recently found to methylate histone 4 at lysine 20 (H4K20) in T lymphocytes.^22^ In addition, SMYD2 could methylate non-histone proteins such as p53, HSP90, RB1, ERα, PARP1, STAT3, NF-κB, and EZH2,^23-30^ and regulate various signaling pathways and cellular events.^17,31^ The downregulation of SMYD2 is linked to adverse cardiac function and increased inflammation, key hallmarks of cardiovascular pathology.^32-34^ Recently, SMYD2 has been implicated in abdominal aortic aneurysm,^35,36^ although the mechanism of its action remains elusive.

In this study, we aimed to explore the role of SMYD2 in phenotypic modulation of VSMCs and vascular intimal hyperplasia. Gain- and loss-of-function *in vitro* and *in vivo* studies revealed that SMYD2 modulated VSMC phenotypic switch and proliferation by direct interaction with myocardin to mediate methylation of H3K4 at CArG regions of VSMC marker gene promoters. Our work establishes SMYD2 as a negative epigenetic regulator of vascular remodeling associated with proliferative vascular diseases featuring dysregulated VSMC phenotype and proliferation, such as restenosis, aneurysm, hypertension, and asthma.

## Materials and methods

### Generation of SMC-specific *Smyd2* knockout mice

*Smyd2*^*tm1a(KOMP)wtsi*^ mice (cryopreserved embryos, RRID: MGI:5781753) were obtained from the Knockout Mouse Project (KOMP) repository at University of California-Davis. Details on the targeted allele are available on the KOMP website and methods used on the CSD targeted alleles have been described before.^37^ The allele contains LoxP elements flanking exon 2 of the *Smyd2* gene and an FRT-flanked selection cassette containing the Engrailed-1 splice acceptor (sA), β-Gal and neomycin selection elements (see Supplementary Figure S1A). Animals were crossed with FlpE recombinase transgenic mice to remove the selection cassette, resulting in the *Smyd2* floxed allele (*Smyd2*^*flox*^). Homozygous *Smyd2*^*fl/fl*^ mice were viable and fertile, and showed no abnormalities compared to fl/wt or wt/wt counterparts. The floxed mutation does not affect SMYD2 expression in homozygous *Smyd2*^*fl/fl*^ mice.

To target *Smyd2* in SMCs, *Smyd2*^*fl/fl*^ mice were first bred with C57BL/6J *Tagln-Cre* transgenic mice (C57BL6.Cg-Tg (*SM22α-Cre*)1Her/J; Stock No: 004746 from Jackson Laboratory, RRID: IMSR_JAX:004746), ^38^ and *Smyd2^fl/+^ Tagln-Cre* mice then crossed to obtain *Smyd2^fl/fl^ Tagln-Cre* animals, i.e., the SMC-specific *Smyd2* knockout mice (denoted as *Smyd2*^*SMC−/−*^). *Smyd2*^*fl/fl*^ homozygous littermates serve as controls (sex ratio per cohort balanced). PCR using tail genomic DNA from 3-week-old offspring confirmed genotype of the mice. Genotyping primers were listed in Supplementary Table S1. All animal protocols were approved by the Institutional Animal Care and Use Committee at Georgia State University in accordance with NIH guidelines.

### Ligation injury of common carotid artery and lentiviral gene transfer via perivascular delivery

Carotid artery ligation injury was induced on male C57BL/6 mice (Jackson Laboratory, 8-10 weeks old) or *Smyd2*^*fl/fl*^ and *Smyd2*^*SMC−/−*^ mice (generated as described above), and the ligation procedure was performed as previously described. ^39,40^ Mice were anesthetized by a single intraperitoneal injection with a mixture of ketamine/xylazine (100 mg/kg body weight and 10 mg/kg body weight, respectively). After the animals were anesthetized, the left common carotid artery (LCA) was dissected and completely ligated near the carotid bifurcation into internal and external carotid artery branches with a 6-0 propylene string through a midline neck incision. A similar procedure was performed (without ligation) on the right common carotid artery (RCA) serving as an uninjured contralateral sham control. The right and left carotid arteries were harvested at various time points, i.e., 7, 14, 21, 28 days after injury, and arterial tissues were processed for either histological examination by hematoxylin and eosin (H&E) staining or Western blot analysis as described below.

For perivascular localized lentivirus delivery in some animals (male C57BL/6 mice at age of 8-10 weeks), following the ligation procedure, the injured artery was gently rinsed with sterile saline and then incubated with GFP control lentivirus or GFP-SMYD2 lentivirus (1 x 10^9^ Tu) for 30 min, packaged by 100 μl Pluronic gel F-127 (Sigma) to extend virus contact time and delivery to bilateral carotid arteries at the time of ligation. The right and left carotid arteries were harvested either 3 or 21 days after injury and processed for Western blot analysis of SMYD2 and GAPDH (3 days post injury) or H&E staining (21 days post injury). The mouse GFP-SMYD2 lentiviral particles (CAT#: MR206911L4V) and GFP control lentiviral particles (CAT#: PS100093V) were purchased from Origene company and the plasmids were sequenced to verify the integrity of the insert.

### Sectioning, H&E (hematoxylin/eosin) staining, and morphometric analysis of intimal hyperplasia

Mice were euthanized by an overdose of CO_2_ inhalation, and common carotid arteries were collected after perfusion fixation with 4% paraformaldehyde (PFA). The collected arteries were further fixed in 4% PFA overnight and then processed for sectioning and morphometric analysis as described below. Common carotid arteries from the ligature to the aortic arch were prepared for cross-sections (5-μm thickness). H&E staining was carried out as described previously ^41^ and digital images of all sections were obtained with a Leica microscope. Sections were analyzed blindly by an independent investigator for neointimal areas and intima-to-media ratios using ImageJ software. The neointimal area was calculated by subtraction of luminal area from the area enclosed by the internal elastic lamina (IEL). The medial area was calculated by subtraction of the area enclosed by the IEL from the area enclosed by the external elastic lamina (EEL). Intimal hyperplasia was quantified as a ratio of neointima area vs media area (intima/media ratio). The data was generated by averaging 5 to 7 sections from each animal, and these sections were located at around 250 μm proximal to the ligature.

### Isolation of mouse primary VSMCs

Primary mouse VSMCs were isolated from aortas of C57BL/6, *Smyd2*^*fl/fl*^ or *Smyd2*^*SMC−/−*^ mice (8-10 weeks old) by enzymatic digestion and cultured as described previously. ^40-43^ Briefly, mouse thoracic aortas were dissected to remove adhering peri-adventitial tissue (surrounding adipose tissues and para-aortic lymph nodes) and the endothelium was denuded with a catheter. Then, the aortic tissues were digested with Collagenase (Sigma, 1 mg/ml) for 10 min at 37°C followed by removing the adventitial layer, and the remaining medial layer was minced into 1-mm small pieces for a second digestion with Collagenase for 2 h at 37°C. After incubation, the digested aortas were rigorously pipetted up and down several times and filtered through a 70-μm cell strainer into 15 ml tubes. Then, the single cell digestion solution was centrifuged (1000 rpm, 5 min, room temperature) to remove the digestion solution. The cells were re-suspended in VSMC culture medium and seeded into 0.1% gelatin-coated culture plates/dishes in Dulbecco’s modified Eagle’s medium (DMEM, Gibco) containing 10% fetal bovine serum (FBS) and 1% penicillin/streptomycin. Every batch of the isolated cells was tested by smooth muscle marker SM α-actin staining to ensure that the purity of primary VSMCs was above 95%. Passages 3-6 of primary mouse VSMCs were used in all experiments unless otherwise specified.

### Cell culture, siRNA transfection, lentiviral transduction, and treatments

Primary human aortic vascular smooth muscle cells (HASMCs; T/G HA-VSMC, #CRL-1999, RRID: CVCL 4009) and C3H/10T1/2 cells (#CCL-226, RRID: CVCL 0190) were obtained from ATCC (Manassas, VA, USA). PAC1 cells (a pulmonary artery-derived SMC line ^44^) were kindly provided by Dr. Li Li from Wayne State University. HASMCs, 10T1/2, PAC1 cells were cultured in high-glucose DMEM with 10% FBS supplemented with penicillin (100 IU/ml) and streptomycin (100 μg/ml) at 37°C in a 5% CO_2_/water-saturated incubator. PAC1 cells were grown in DMEM/10% FBS until sub-confluency, then washed in sterile phosphate-buffered saline (PBS) three times and switched to serum starvation (0.5% FBS) for 24-48 h as the differentiated condition, before treated with PDGF-BB (10-20 ng/ml) or SMYD2 inhibitor (Bay-598). HEK293T cells (obtained from ATCC, RRID: CVCL 0063) were cultured in DMEM with 10% FBS as above for lentivirus production or transient transfections. Both murine and human VSMCs between passages 3 - 8 were used in all experiments.

For siRNA transfection, cells in 6-well plate at 80% confluency were transfected with siRNA duplex against SMYD2 or myocardin, or scrambled control siRNA duplex using MISSION siRNA Transfection Reagent (Sigma) in Opti-MEM medium (Gibco) according to manufacturer’s instructions. After transfection for 48 h, cells were serum starved (0.5% FBS) for 24 h (or overnight), followed by treatments and assays as described below. Lentiviral transduction was performed as per the manufacturer’s instructions. Briefly, low passage (P3-5) VSMCs at approximately 80% confluency were infected with lentiviral particles carrying either GFP control vector or GFP-SMYD2 overexpressing vector for 24-48 h, and the clones expressing SMYD2 were selected in media containing puromycin dihydrochloride (2 μg/ml) to screen for stable transduction. Upregulation of SMYD2 was evaluated by qPCR and Western blot analysis.

For SMYD2 inhibition study, cells were serum-starved (0.5% FBS) for 24 h and then treated with SMYD2 specific inhibitor (Bay-598) ^45,46^ at various concentrations (0 - 5 μM) in low serum (0.5% FBS) medium for 24-48 h, before proceeded for cell proliferation and migration assays as well as visualization of SMC marker proteins via immunofluorescence microscopy as described below. For PDGF-BB treatment experiments, VSMCs were grown to 80-90% confluence and serum-starved (0.5% FBS) for 24 h, followed by incubation with 20 ng/ml PDGF-BB for 6, 12, 24, and 48 h. Cells treated with vehicle served as control. For cells in some experiments, when necessary, media and treatments were refreshed every 24–48 h. Following PDGF-BB or vehicle administration, cells were processed for functional assays or harvested for detection of mRNA or protein expression by qRT-PCR or Western blotting, respectively, as described below.

### SMYD2 knockout via CRISPR/CAS9 in PAC1 cells

Rat *Smyd2*-specific CRISPR single guide RNAs (sgRNAs) were designed on a website (http://crispr.mit.edu/) with a view to their uniqueness and off-target effects, and then were purchased from Sigma. Subsequently, sgRNAs were annealed and cloned into the pSpCas9(BB)-2A-Puro plasmid (pX459, Feng Zhang Laboratory, Addgene #62988, RRID:Addgene_62988) according to published protocol. ^47^ The pX459 plasmid expresses a human codon-optimized SpCas9 in addition to the guide RNA and puromycin resistance for cell selection. The CRISPR-Cas9 plasmids were confirmed by sequencing. The sgRNA sequences are listed in Supplementary Table S2.

### Immunofluorescence staining and microscopy

VSMCs were seeded on glass coverslips in 24-well cell culture plates at a density of 10^3^ cells/cm^2^, and then incubated overnight in normal cell growth conditions. For immunofluorescence staining, cells were washed with PBS for 3 times followed by fixation and permeabilization in 75% acetone in ethanol for 5 min. Cells were then blocked with 10% goat serum for 1 h and incubated overnight with primary antibodies against SMYD2 (# 9734, Cell Signaling, RRID: AB 10889559), SM α-actin (ab5694, Abcam, RRID: AB 2223021), myocardin (sc-33766, Santa Cruz, RRID: AB 2148748), myocardin-related transcription factor-A (MRTF-A: sc-398675, Santa Cruz, RRID: AB 2142498), and MRTF-B (PA5-37105, Thermo Fisher Scientific, RRID: AB 2553910). Cells were then rinsed with PBS for 3 times and incubated with Alexa Fluor 488 or 568-conjugated secondary antibodies (1:400 dilution, Thermo Fisher). Finally, cells were counter-stained with DAPI (1:10,000 dilution, Sigma) and mounted with mounting medium. Fluorescence images of stained cells were captured via using a Leica immunofluorescence microscope.

### Quantitative reverse transcription-PCR (RT-qPCR) analysis

Total RNA was extracted from cells using TRIzol reagent (AM9738, Invitrogen) according to the manufacturer’s instruction and cDNA was synthesized by High Capacity RNA-to-cDNA Kit (Applied Biosystems). qPCR was performed by using SYBR Green master mix (Thermo Fisher). Relative gene expression was converted using the 2^−ΔΔCT^ method against the internal control housekeeping gene GAPDH, where ΔΔCT = (CT _experiment gene_ - CT _experiment GAPDH_) - (CT _control gene_ - CT _control GAPDH_). The relative gene expression in control groups was set to 1. The qPCR primer sequences used in this study were listed in Supplementary Table S3. All samples were amplified in triplicate and all experiments were repeated independently at least 3 times.

### Protein extraction and Western blot analysis

Western blot analysis was performed according to standard procedures using lysates from tissues or cells as we described previously. ^48-50^ Briefly, cells were lysed with M-PER Mammalian Protein Extraction Reagent (78501, Thermo Fisher) supplemented with the protease inhibitor cocktail (P8340, Sigma) for 30 min on ice and then cell lysates were centrifuged at 13,000 g for 15 min. Mouse carotid arteries were harvested by cutting open longitudinally and removing off the adventitia as described previously. ^40,42^ Tissues were cut into small pieces and ground with a glass homogenizer in Extraction buffer as above. After sonication and centrifugation of the lysates, proteins were quantified by Bradford method. Equal amounts (30-40 μg) of total proteins from each sample were resolved on SDS-PAGE, and transferred to PVDF membranes (#1620177, Bio-Rad). The membranes were blocked with 5% goat serum at room temperature for 1 h before the addition of primary antibodies (as listed below) for overnight incubation at 4°C, followed by incubation with HRP-conjugated secondary antibodies (Sigma) for 1 h at room temperature. The ECL chemiluminescent kit was used for signal detection, and the images were captured using Amersham Imager 600 RGB system (GE healthcare). The primary antibodies against the following proteins were used: SMYD2 (sc-393827, Santa Cruz; or #9734, Cell Signaling, RRID: AB 10889559), SM α-actin (ab5694, Abcam, RRID: AB 2223021), SM22α (ab14106, Abcam, RRID: AB_443021), SM-MHC (ab133567, Abcam, RRID: AB 2890982), myocardin (sc-33766, Santa Cruz, RRID: AB 2148748; or SAB4200539, Sigma), SRF (sc-335, Santa Cruz, RRID: AB 2255249), HA (H9658, Sigma, RRID: AB 260092), GST (#05-782, Sigma), GAPDH (AM4300, Ambion, RRID: AB 437392).

### Cell proliferation assays via using CCK8 and cell counting

VSMC proliferation was measured *in vitro* using a Cell Counting Kit-8 (CCK8; Dojindo, Kumamoto, Japan) according to manufacturer’s instructions as we described previously. ^48^ In brief, following gene knock-down by siRNA transfection or overexpression by lentiviral transduction as described above, VSMCs were equally re-plated into 96-well plates at a density of 1×10^3^ cells/well, and maintained in 0.5% FBS-containing DMEM medium for 24 h to induce growth arrest. Cells were then stimulated with 10 ng/ml PDGF-BB for 24 h at 37°C. Afterwards, the rate of cell proliferation was determined by incubation with 10 μl CCK-8 solution per well for additional 2 h at 37°C, and the absorbance at 450 nm, as an indirect measure of cell proliferation, was measured using a microplate reader.

For cell number counting assays, primary mouse aortic VSMCs or cultured VSMCs after SMYD2 knock-down or overexpression and/or myocardin knock-down were equally seeded onto 6-well plates and serum starved for 24 h, before cells were stimulated with 10 ng/ml PDGF-BB for 24 h. The cells were then trypsinized and the changes in cell counts were determined manually using a hemocytometer as reported previously. ^41^ All samples were run in triplicate.

### Transwell migration assays

VSMC migration was measured using a modified Boyden chamber assay as described previously. ^40,42,48,51^ Briefly, primary mouse aortic VSMCs or cultured VSMCs (PAC1, HASMCs) were subjected to siRNA transfection, lentiviral transduction, or SMYD2 inhibition, followed by serum starvation for 24 h, as described above. Then, 2.5 x 10^4^ cells in 100 μl serum-free medium were added to the top chambers of a 24-well Transwell plate (8-μm pores; Corning, NY), and 500 μl medium with or without PDGF-BB (10 ng/ml) was added to the lower chamber. After 16 h culture in a 37°C incubator, the top-side of the membranes was swabbed to remove cells, and cells on the bottom surface were fixed with cold methanol and stained with 0.1% crystal violet. Five randomly chosen high-power fields (HPF) per membrane were used to count the average number of migrated cells. The cell numbers on each field were counted by Image J software (NIH).

### Whole transcriptome RNA sequencing (RNA-seq)

Differential gene expression analysis was performed using RNA sequencing at the BGI company (Cambridge, MA). After isolation of primary VSMCs from aortas of *Smyd2*^*fl/fl*^ and *Smyd2*^*SMC−/−*^ mice, VSMCs (at passage 4) were starved overnight and then harvested to extract total RNA by TRIzol (Invitrogen) for whole transcriptome RNA-seq analysis as described before. ^41^ The first step in the transcriptome experimental workflow involves purifying the poly-A containing mRNA molecules using poly-T oligo attached magnetic beads. Following purification, the mRNA was fragmented into small pieces using divalent cations under elevated temperature. The cleaved RNA fragments were copied into first strand cDNA using reverse transcriptase and random primers, followed by second strand cDNA synthesis using DNA Polymerase I and RNase H. These cDNA fragments then have the addition of a single 'A' base and subsequent ligation of the adapter. The products were then purified and enriched with PCR amplification. PCR yield was then quantified by Qubit and samples were pooled together to make a single strand DNA circle (ssDNA circle), which gave the final library. DNA nanoballs (DNBs) were generated with the ssDNA circle by rolling circle replication (RCR) to enlarge the fluorescent signals at the sequencing process. The DNBs were loaded into the patterned nanoarrays and pair-end reads of 100 bp were read through on the BGISEQ-500 platform for the following data analysis. For this step, the BGISEQ-500 platform combines the DNA nanoball-based nanoarrays and stepwise sequencing using Combinational Probe-Anchor Synthesis (cPAS) sequencing method. After filtering with internal software SOAP nuke, the remaining reads are called "Clean Reads" and stored in FASTQ format followed by mapping step with HISAT software. Clean reads were mapped to reference using Bowtie2, and gene expression level was then calculated with RSEM. Differential gene expression analysis was performed at the BGI company (Cambridge, MA). Cutoff values of fold change greater than 2 and FDR less than 0.01 were considered significant between *Smyd2*^*fl/fl*^ VSMCs and *Smyd2*^*SMC−/−*^ VSMCs. Counts per million (CPM) was calculated from raw counts and used to quantify relative expression of genes for each sample. The hierarchical clustering for differentially expressed genes (DEGs) was performed using pheatmap, a function of R. For clustering more than two groups, the intersection and union DEGs between groups were performed. Volcano plots were generated using R program. Gene Ontology (GO) analysis was carried out by g:Profiler. Heat map was generated by MeV (MultiExperiment Viewer) program. The RNA-seq data generated in this study have been deposited in the Gene Expression Omnibus (GEO) at the NCBI under accession number GSE161004.

### Co-immunoprecipitation (co-IP) assays

To determine if SMYD2, myocardin, and/or p300 can form a complex in cells, 10T1/2 cells or HEK293T cells were co-transfected with expression plasmids encoding Myc-tagged SMYD2, Flag-tagged myocardin, and/or HA-tagged p300 with Lipofectamine 3000 (ThermoFisher) for 48 h. Subsequently, nuclear protein was harvested for coimmunoprecipitation (co-IP) assays using the Nuclear Complex Co-IP Kit (#54001, Active Motif) according to the manufacturer’s protocol as we previously reported. ^43^ Equal amounts (300 μg) of nuclear protein extracts from each sample were incubated with 3 μg of each primary antibody: anti-Flag antibody (F7425, rabbit polyclonal, Sigma, RRID: AB 439687), anti-Myc antibody (ab9106, rabbit polyclonal, Abcam, RRID: AB 307014), and rabbit normal IgG (#2729, rabbit, Cell Signaling, RRID: AB 1031062), in 500 μl low salt IP buffer (Active Motif) with gentle rotation overnight at 4°C. The lysates immunoprecipitated with normal rabbit IgG served as negative control. Afterwards, 20 μl protein A/G beads (sc-2003, Santa Cruz, RRID: AB 10201400) were added to the lysates and incubated for 3 h at 4°C to pull-down the immobilized protein complexes. The beads with bound proteins were washed 3 times with IP wash buffer and resuspended in sample buffer and boiled at 95°C for 5 min. The immunoprecipitated proteins were further analyzed by Western blotting as described above, using the following primary antibodies: anti-SMYD2 (sc-393827, mouse monoclonal, Santa Cruz), anti-myocardin (SAB4200539, rabbit polyclonal, Sigma), and anti-HA (H9658, mouse monoclonal, Sigma, RRID: AB 260092).

### Protein purification and GST pull-down assays

To further characterize the interaction between SMYD2 and myocardin *in vitro,* various fragments of each of the proteins were expressed as fusion proteins in bacteria. Bacterial expression of full-length proteins (mouse myocardin, and SMYD2) and truncated proteins (mouse myocardin truncation mutants) and GST pull-down assays were performed essentially as described in our previous reports. ^43,50,52,53^ pGEX-4T vectors (express GST fusion protein, Sigma-Aldrich) containing full-length or truncated mouse myocardin cDNA were generated as previously reported. ^53^ pET30 vector (express His and S fusion protein, Novagen) containing full-length SMYD2 cDNA was generated as we described before. ^50,52^ Six myocardin deletion mutants were expressed as GST fusion proteins: (1) NT-myocardin, encoding amino acids 1-585; (2) CT-myocardin, encoding amino acids 585-935; (3) N1-myocardin, encoding amino acids 1-220; (4) N2-myocardin, encoding amino acids 220-350; (5) N3-myocardin, encoding amino acids 350-474; and (6) N4-myocardin, encoding amino acids 474-585. In brief, bacterial expression vectors were transformed into *Escherichia coli* BL21-star (Stratagene) cells. After 1 h of induction with 0.4 mM isopropyl β-D-thiogalactoside (IPTG), cultured bacteria were harvested by centrifugation, resuspended in PBS with protease inhibitors and then lysed by sonication. The His-S fusion protein (SMYD2) in pET30 vector were purified using Talon beads (binding to His tag) and eluted with 200 mM imidazole as described before. ^52^ The GST fusion protein lysates were clarified by centrifugation and incubated for 1 h with a 50% suspension of glutathione-agarose beads (Amersham Biosciences) in PBS. Beads bound with GST fusion proteins (GST-myocardin full-length or GST-myocardin truncation fusion proteins) were resuspended and incubated for 2 h with His-SMYD2 full-length fusion protein in a total volume of 1 ml of binding buffer (PBS containing 1% Triton X-100, 1 mg/ml BSA with protease inhibitors). After the beads were washed 3 times in 1 ml of washing buffer (PBS containing 1% Triton X-100), the bound proteins were eluted by heating at 95°C for 5 min in SDS sample buffer. The eluted GST and His fusion proteins were resolved on SDS-PAGE and detected by Western blotting as described above, using anti-SMYD2 antibody (sc-393827, Santa Cruz), and membrane was stripped and re-probed with anti-GST antibody (#05-782, Sigma) to check GST fusion proteins. Images were captured using Amersham Imager 600 RGB system (GE healthcare).

### In situ proximity ligation assays (PLA)

Primary murine aortic VSMCs were cultured in Lab-Tek chamber slides and then serum-starved for 24 h, before treated with PDGF-BB (10 ng/ml) or vehicle control for 24 h. Thereafter, cells were washed in PBS, fixed in 25% acetone and 75% ethanol for 15 min. The proximity ligation assays (PLAs) were performed according to the manufacturer’s instructions with minor modifications, as described previously. ^54^ Briefly, primary antibodies, anti-SMYD2 (sc-393827, mouse monoclonal, Santa Cruz) and anti-myocardin (sc-33766, rabbit polyclonal, Santa Cruz, RRID: AB_2148748) were incubated with cells followed by combinations of corresponding PLA probes (i.e., anti-rabbit PLUS, anti-mouse MINUS PLA probes) for 1 h at 37°C. Subsequent ligations and detections were performed by using DuoLink II Detection Reagents Orange Kit (OLINK Bioscience). All the steps, including blocking, antibody hybridization, proximity ligation, and detection (excitation/emission: 554/579 nm), were conducted following the manufacturer’s instructions (Duolink IQ, OLINK Bioscience). The nuclei were stained using DAPI. Cells were mounted in DuoLink mounting medium (OLINK Bioscience) for visualization and image capture by a Leica microscope. Quantification of proximity ligation signal was performed using ImageJ. Each individual data point represents an average of 4 fields of view from 3 slides with at least 50 cells per field of view. Data were quantified as ratio of PLA punctates to number of nuclei per sample.

### Chromatin immunoprecipitation-quantitative PCR (ChIP-qPCR) assays

Chromatin immunoprecipitation - quantitative PCR (ChIP-qPCR) was performed as we reported previously ^43^ and described by others ^9,55^ with slight modifications. For the ChIP assays, VSMCs in culture plates (with or without lentiviral transduction or siRNA transfection, or PDGF-BB treatment) were cross-linked with 1% formaldehyde at room temperature for 10 min, and then washed with ice-cold PBS and resuspended in lysis buffer (50 mM Tris-HCl, pH 8.0, 0.5% SDS, 5 mM EDTA). DNA fragments were generated by sonication of cross-linked chromatin nuclear extracts with Sonifier^®^ SFX250 (BRANSON) for 1-3 min, followed by immunoprecipitation using the following ChIP grade antibodies: normal rabbit IgG (#2729, Cell Signaling, RRID: AB 1031062) or normal mouse IgG (#12-371, Sigma, RRID: AB 145840), anti-SMYD2 (ab108217, Abcam, RRID: AB 10863887), anti-myocardin (SAB4200539, Sigma), anti-SRF (sc-25290, Santa Cruz, RRID: AB 2239787), anti-H3K4me1 (ab8895, Abcam, RRID: AB 306847), anti-H3K4me2 (ab7766, Abcam, RRID: AB 2560996), anti-H3K4me3 (ab8580, Abcam, RRID: AB 306649). The antibody-antigen complexes were incubated overnight at 4°C with gentle rotation, and then Protein G Dynabeads (Invitrogen) were added and rotated for another 2 h. The immunoprecipitates were eluted from the beads and treated with protease K to digest proteins. Immunoprecipitated DNA was then extracted with phenol-chloroform, ethanol precipitated and eluted. The DNA purified from the immunoprecipitated genomic DNA or from input were amplified by qPCR using specific primers for the CArG regions of *SM α-actin* and *SM-MHC* promoters (listed in Supplementary Table S4). Data were expressed as relative binding/enrichment by using the 2^−ΔΔCT^ method against the control samples (set to 1). All samples were performed in triplicate, and data were normalized to % input.

### Transient transfection and luciferase reporter assays

Transient transfection and reporter gene luciferase assays were carried out as previously described. ^43,56^ Briefly, 10T1/2 cells or HEK293T cells were maintained in DMEM/10% FBS with 1% penicillin and streptomycin. Then the cells were seeded in 12-well plates one day before transient transfection. Sub-confluent cells were co-transfected using Lipofectamine 3000 (ThermoFisher) with the luciferase reporter construct (in pGL3B vector, Promega) harboring *SM a-actin* gene promoter (nucleotide positions −2555 to +2813 containing CArG regions as reported before ^57^), CMV-Renilla luciferase reporter as an internal control (Promega), along with or without SMYD2 and/or myocardin expression plasmids. Post-transfection 24-48 h, cell lysates were collected, and the luciferase activity was determined using the Dual-Luciferase Reporter Assay System kit (Promega) by a plate reader according to the manufacturer’s instruction. The firefly luciferase activity values in the lysates were normalized to the TK Renilla luciferase reporter activity as the internal control for transfection efficiency. The data were presented as the fold changes relative to empty vector or myocardin alone (set to 1). A minimum of 3 independent transfections was performed and all assays were replicated at least three times.

### Statistical analysis

All data are expressed as mean ± SEM of at least three independent experiments. Data analysis was performed by using GraphPad Prism 8 program (GraphPad Software, Inc, La Jolla, CA). An unpaired, two-tailed Student’s t-test was used to analyze the difference between two groups of data with normally distributed variables. Differences across three or more groups were determined with one-way analysis of variance (ANOVA) followed by Bonferroni’s post hoc test. Mann–Whitney test was used in non-normally distributed variables, where appropriate. A *P* value of less than 0.05 was considered significant.

## Results

### SMYD2 expression is down-regulated in injury-induced neointima in mice and phenotypically modulated VSMCs

**Figure 1.**
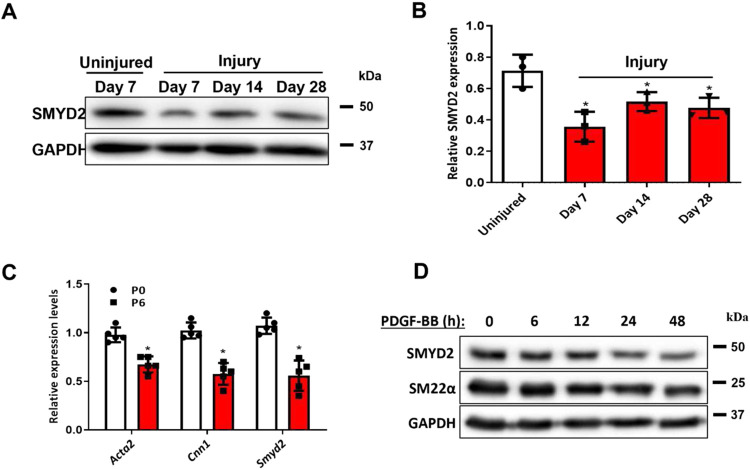
SMYD2 expression was down-regulated in injured mouse carotids and phenotypically modulated VSMCs in vitro. **A,** SMYD2 expression in carotid arteries of C57BL/6 mice following ligation injury of left common carotid arteries (LCA) for 7, 14 and 28 days. The uninjured right carotid arteries (RCA) were used as controls. **B,** Densitometric analysis of SMYD2 expression in mouse carotid arteries normalized to GAPDH (n=3 independent experiments). **C,** Expression of *Smyd2* and SM marker genes (*Acta2* and *Cnn1*) was determined by RT-qPCR in different passages (P0 and P6) of primary mouse aortic VSMCs cultured in 10% FBS + DMEM, values were normalized to *β-actin* (n=5 independent experiments). **D,** Western blot analysis of SMYD2 and SM22α in human VSMCs (HASMCs) upon treatment with PDGF-BB (20 ng/ml) for various durations (0-48 h), followed by serum starvation for 24 h. GAPDH served as the loading control. For all bar graphs, data are expressed as the means ± SEM, *P < 0.05 (unpaired, two-tailed Student’s t-test).

Mechanical injury to arteries induced phenotypic switching of medial layer VSMCs accompanied with increased VSMC proliferation and migration, which culminated in intimal hyperplasia.^58^ To determine whether SMYD2 is involved in SMC phenotypic modulation and vascular remodeling post-arterial injury in mice, we performed the carotid artery ligation, a model of vascular injury that induces a SM-rich neointima and remodeling,^59^ and then examined SMYD2 expression in carotid arterial tissues. Western blot assays revealed a significant downregulation of SMYD2 expression in injured carotid arteries as shown that SMYD2 protein levels were drastically decreased at day 7 after injury, then restored to some extent but still maintained lower levels than controls at days 14 and 28 after injury (Figure 1A and B).

The phenotypic switch of VSMCs from a contractile phenotype to a synthetic state is involved in various vascular diseases including aneurysm, hypertension, and restenosis.^2,3,13^ Next, we wondered whether SMYD2 expression is modulated in VSMC phenotypic switch *in vitro.* Generally, early-passage VSMCs represent a differentiated state, whereas late-passage VSMCs exhibit a less differentiated phenotype.^60^ As expected, expression levels of SMC marker genes *Acta2* and *Cnn1* as well as SMYD2 in primary murine VSMCs were significantly downregulated at later passage (P6) compared with early passage (P0) (Figure 1C). Furthermore, SMYD2 expression in cultured human aortic smooth muscle cells (HASMCs) was substantially downregulated in a time-dependent manner upon treatment with PDGF-BB, which is known to induce phenotypic switch by inhibiting SMC markers such as SM22α (Figure 1D). Collectively, these data demonstrate that SMYD2 expression is downregulated in murine arterial injury *in vivo* and during VSMC phenotypic switching *in vitro.*

### *Smyd2* deletion in VSMCs aggravates while its overexpression attenuates injury-induced neointima formation in mice

**Figure 2.**
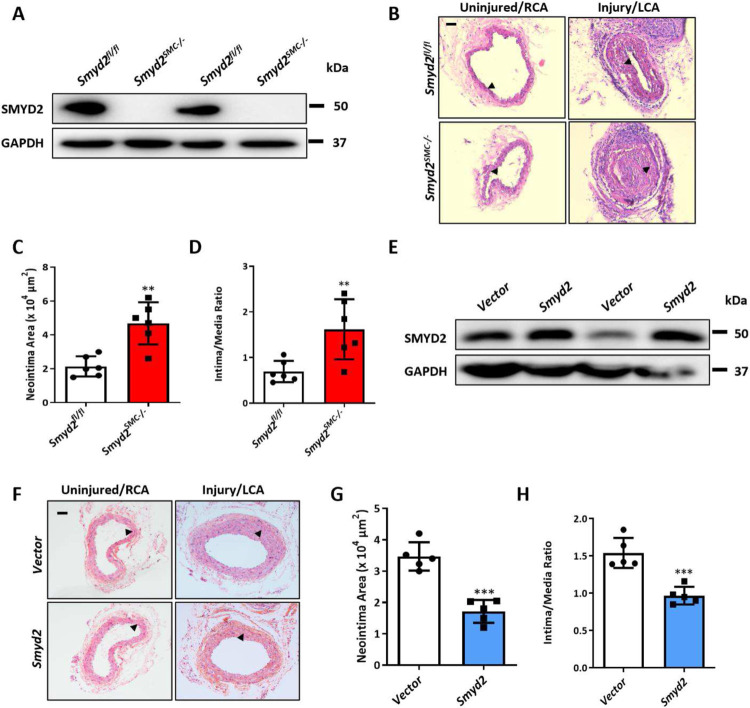
SMC-SMYD2 regulated neointima formation in the carotid arteries after injury in mice. **A,** Western blot analysis of SMYD2 protein in primary aortic VSMCs isolated from *Smyd2^fl/fl^* control mice and *Smyd2*^*SMC−/−*^ mice. **B,** Representative images of H&E-stained cross-sections of carotid arteries from *Smyd2*^*fl/fl*^ control mice and *Smyd2*^*SMC−/−*^ mice following sham or ligation injury for 14 days. Arrows indicated internal elastic lamina (IEL), Scale bar: 100 μm. **C** and **D**, Quantitative analysis of neointima area (**C**) and intima-to-media ratio (**D**) at day 14 post-injury (n=6 mice per group). **E,** GFP vector or GFP-SMYD2 lentiviruses (1×10^9^) were injected immediately around sham-operated or injured carotid arteries in C57BL/6 mice. Expression of SMYD2 and GAPDH in carotid arteries of GFP vector or GFP-SMYD2 group 3 days post-injury/injection was examined by Western blotting. **F,** Representative images of H&E-stained sections of carotid arteries from C57BL/6 mice locally injected with GFP vector control or GFP-SMYD2 overexpression lentivirus, at day 21 post-injury. Arrows indicated IEL. Scale bar: 100 μm. **G** and **H**, Quantitative analysis of neointima area (**G**) and intima-to-media ratio (**H**) at day 21 post-injury (n=5 mice per group). For all bar graphs, data are expressed as the means ± SEM, **P < 0.01, ***P < 0.001 and ****P < 0.0001 (unpaired, two-tailed Student’s t-test).

To investigate the *in vivo* function of SMC-expressed SMYD2, and to evaluate whether the loss of *Smyd2* could be a cause or a consequence of intimal hyperplasia following vascular injury, we generated SMC-specific *Smyd2*-deficient (*Smyd2*^*SMC−/−*^) mice by breeding *Smyd2* homozygous floxed mice with *SM22α-Cre* transgenic mice (Supplementary Figure S1A). *Smyd2*^*SMC−/−*^ mice were born at normal Mendelian frequencies and developed normally to adulthood. The mice were genotyped by RT-PCR (Supplementary Figure S1B) and specific deletion of *Smyd2* gene in VSMCs was confirmed by Western blot analysis (Figure 2A, Supplementary Figure S1C). We next performed the carotid artery ligation injury in *Smyd2*^*SMC−/−*^ and control mice. Histological analysis demonstrated that *Smyd2* ablation in VSMCs accelerated neointima formation after 14-day injury as shown by the larger neointima size and significantly increased intima-to-media ratio compared to the control *Smyd2*^*fl/fl*^ group (Figure 2B-D). These findings suggest that *Smyd2* deficiency in VSMCs renders mice more susceptible to neointima formation after vascular injury.

To determine whether local administration of exogenous SMYD2 mitigates injury-induced intimal hyperplasia, *Smyd2* lentivirus or control lentivirus was peri-vascularly applied to carotid arteries of C57BL/6 mice immediately after ligation injury as described previously.^40^ Western blot analysis of carotid arteries showed that SMYD2 was significantly upregulated after the delivery of *Smyd2* lentivirus compared with control virus (Figure 2E). *Smyd2* lentivirus-infected carotid arteries had attenuated neointima formation 3 weeks after ligation injury, as shown by significantly decreased neointima area (Figure 2F-G) and intima/media ratio (Figure 2H), indicating that SMYD2 protects against injury-induced intimal hyperplasia. Taken together, these results suggest that deletion of *Smyd2* in VSMCs aggravates, while overexpression of *Smyd2* attenuates arterial injury-induced neointima formation in Mice.

### SMYD2 inhibits VSMC proliferation and migration in vitro

**Figure 3.**
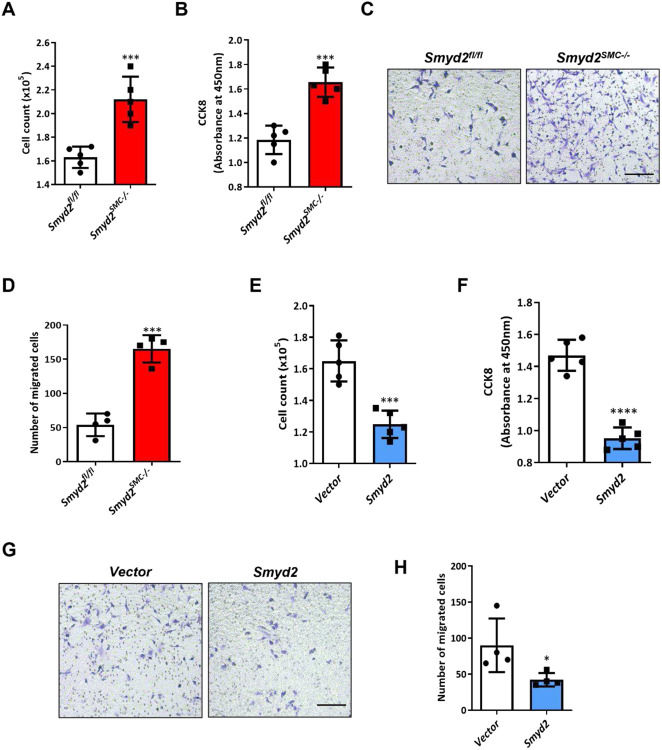
SMYD2 inhibited VSMC proliferation and migration *in vitro*. **A** and **B**, Primary aortic VSMCs isolated from *Smyd2*^*fl/fl*^ or *Smyd2*^*SMC−/−*^ mice were serum-starved for 24 h, and then treated with PDGF-BB (10 ng/mL) for 24 h for the cell counting analysis (**A**) and CCK8 proliferation assay (**B**) (n=5 independent experiments). **C,** Transwell migration assay of murine aortic VSMCs from *Smyd2*^*fl/fl*^ or *Smyd2*^*SMC−/−*^ mice upon stimulation with PDGF-BB for 16 h. Scale bar, 100 μm. **D,** Quantification of migrated murine VSMCs (n=4 independent experiments). **E** and **F**, Primary C57BL/6 mouse aortic VSMCs were transduced with lentivirus encoding *GFP-SMYD2* or *GFP* vector control. Post-transduction 48 h, cells were serum starved for 24 h, followed by PDGF-BB treatment. Proliferation of the transduced VSMCs was determined by cell counting analysis (**E**) and CCK8 proliferation assay (**F**) upon treatment with PDGF-BB for 24 h (n=5 independent experiments). **G,** Transwell migration assay of C57BL/6 mouse aortic VSMCs upon stimulation with PDGF-BB for 16 h. Scale bar, 200 μm. **H,** Quantification of migrated murine VSMCs. (n=4 independent experiments). For all bar graphs, data are expressed as the means ± SEM, *P < 0.05, ***P < 0.001 and ****P < 0.0001 vs. *Smyd2*^*fl/fl*^ or vector group (unpaired, two-tailed Student’s t-test).

VSMC proliferation and migration are two important cellular events leading to neointima formation *in vivo*.^41^ We next directly assessed the effects of SMYD2 on SMC proliferation and migration *in vitro*. We isolated primary VSMCs from *Smyd2^SMC−/−^* mice and *Smyd2^fl/fl^* control mice and treated them with PDGF-BB, a growth factor known to inhibit SMC contractile markers (such as SMA) and promote SMC proliferation and migration.^61,62^ The results of cell counting, CCK8 proliferation assay, and Transwell migration assay demonstrated that *Smyd2* ablation in VSMCs significantly promoted PDGF-BB induced cell proliferation and migration (Figure 3A-D). By loss-of-function assays including CRISPR-Cas9-mediated knockout and transfection with silencing RNA against SMYD2 in rat and human VSMCs, respectively, we observed increased proliferation and migration of VSMCs induced by PDGF-BB (Supplementary Figure S2, S3). Moreover, pharmacological inhibition of SMYD2 methyltransferase activity with SMYD2- specific inhibitor (BAY-598) ^45,46^ promoted PDGF-BB-induced VSMC proliferation and migration (Supplementary Figure S4), suggesting that SMYD2 inhibits VSMC proliferative and migratory activities in an enzymatic activity dependent manner.

As ectopic expression of SMYD2 suppressed vascular intimal hyperplasia *in vivo* (Figure 2F-H), we next assessed the effects of SMYD2 overexpression on VSMC proliferation and migration *in vitro* by transducing GFP-SMYD2 lentivirus into VSMCs. Increased SMYD2 expression was confirmed in GFP-SMYD2 lentivirus transduced VSMCs compared with GFP-vector transduced VSMCs (Supplementary Figure S5). The results of cell counting (Figure 3E), CCK8 proliferation assay (Figure 3F), and Transwell migration assay (Figure 3G, H) showed that overexpression of SMYD2 significantly decreased VSMC proliferation and migration. Collectively, these results demonstrate that SMYD2 inhibits VSMC proliferation and migration, thereby attenuating vascular intimal hyperplasia.

### SMYD2 Promotes VSMC marker gene expression and modulates VSMC phenotypic switch

**Figure 4.**
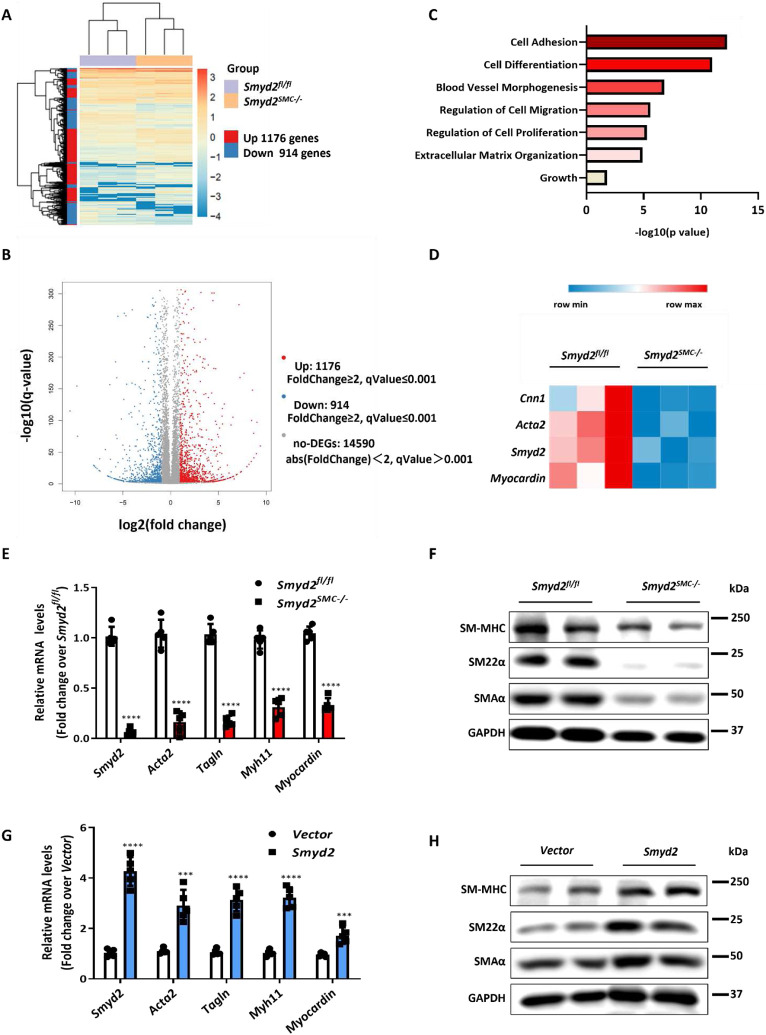
SMYD2 promoted VSMC marker expression and modulated VSMC phenotypes **A,** Hierarchical clustering heat map of mRNAs differentially expressed between primary aortic VSMCs isolated from *Smyd2*^*fl/fl*^ control mice and *Smyd2*^*SMC−/−*^ mice, with cutoff of FC (fold change) ≥2 and adjusted P value ≤0.001; n=3 each group. **B,** Volcano plot, with light blue dots representing significantly downregulated protein-coding genes (n=914) and red dots representing significantly upregulated protein-coding genes (n=1176) in primary aortic VSMCs of *Smyd2*^*SMC−/−*^ mice (q Value = adj *P* value). **C,** Gene ontology (GO) analysis of biological functions related with the differentially expressed genes in *Smyd2*-deficient murine VSMCs. Significant GO terms enriched in differentiated genes were plotted based on P values. **D,** Heat map of significantly downregulated contractile genes in Smyd2-null VSMCs compared with control VSMCs; (n=3 each group). RT-qPCR analysis (n=5 independent experiments) (**E**) and Western blot analysis (**F**) of SMC markers and myocardin in primary aortic VSMCs isolated from *Smyd2*^*fl/fl*^ or *Smyd2*^*SMC−/−*^ mice. RT-qPCR analysis (n=5 independent experiments) (**G**) and Western blot analysis (**H**) of SMC contractile proteins in primary VSMCs transduced with *GFP* or *GFP-Smyd2* lentivirus. For all bar graphs, data are expressed as the means ± SEM, ***P < 0.001 and ****P < 0.0001 (unpaired, two-tailed Student’s t-test).

To elucidate molecular mechanism underlying the function of SMYD2 in VSMCs, we assessed changes in gene expression in VSMCs isolated from *Smyd2*^*SMC−/−*^ or *Smyd2*^*fl/fl*^ mice by RNA sequencing.^41,43^ RNA-seq data demonstrated that ablation of *Smyd2* in VSMCs significantly downregulated 914 genes while upregulated 1176 genes (Figure 4A, B), without affecting the expression of other SMYD family members *Smyd1, Smyd3, Smyd4* and *Smyd5.* Gene Ontology (i.e., biological processes) analysis revealed significant enrichment of genes involved in cell differentiation and adhesion, regulation of cell proliferation and migration, blood vessel morphogenesis, extracellular matrix organization (Figure 4C). After *Smyd2* ablation, several SMC contractile marker genes such as *Acta2, Cnn1*, *Myh11,* and the key SMC determinant *myocardin* were significantly downregulated (Figure 4D). RT-qPCR and Western blot analysis confirmed the RNA sequencing results at both mRNA (*Acta2, Tagln, Myh11, Myocardin*) and protein levels (SM-MHC, SM22α, SMAα) (Figure 4E-F). Conversely, overexpression of *Smyd2* in VSMCs promoted the expression of SMC marker genes and myocardin (Figure 4G-H). However, we did not observe significant change in mRNA or protein levels of myocardin-related transcription factor (MRTF)-A and MRTF-B in *Smyd2* deficient VSMCs, compared to control cells (Supplementary Figure S6). Taken together, these findings indicate that SMYD2 is essential to promoting gene expression of myocardin and VSMC contractile proteins.

### Regulation of VSMC phenotypic switch, proliferation and migration by SMYD2 is myocardin dependent

**Figure 5.**
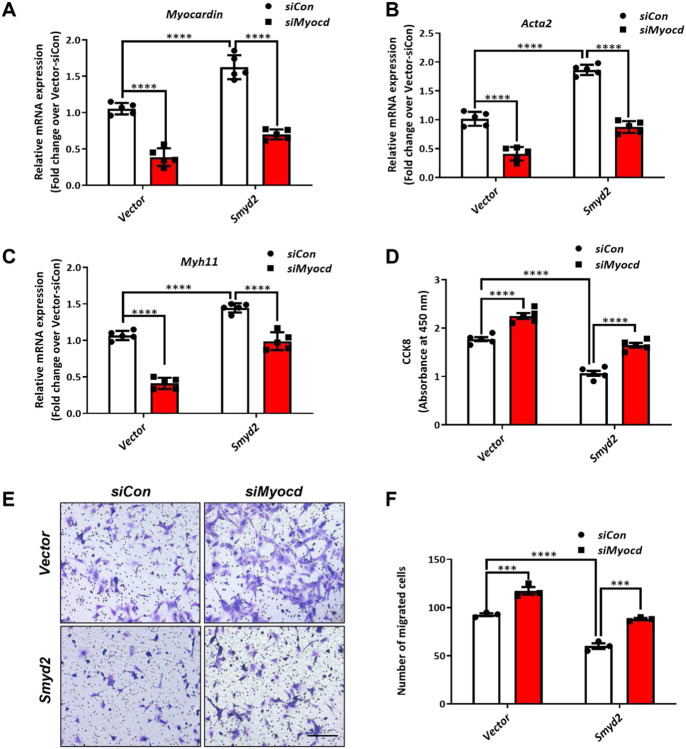
SMYD2-mediated effects on VSMC phenotype was myocardin dependent WT mouse primary aortic VSMCs stably transduced with *GFP* vector control lentivirus or *GFP-Smyd2* lentivirus were transiently transfected with control siRNA (*siCon*) or myocardin siRNA (*siMyocd*) for 48 h. The cells were harvested for RT-qPCR analysis of mRNA expression of *myocardin* (n=5 independent experiments) (**A**), *Acta2* (n=5 independent experiments) (**B**) and *Myh11* (n=5 independent experiments) (**C**). VSMCs were further serum starved for 24 h and then treated with PDGF-BB for CCK8 assay (n=5 independent experiments) (**D**) and Transwell migration assay (**E**, **F**). Scale bar, 200 μm (n=3 independent experiments). For all bar graphs, data are expressed as the means ± SEM, ***P < 0.001 and ****P < 0.0001 (two-way ANOVA with Bonferroni’s post hoc test).

Myocardin is a transcription co-activator that binds to SRF in the CArG regions in SM marker gene promoters to promote SMC contractile gene expression while inhibit VSMC phenotypic switch, proliferation and migration.^6^ Given that SMYD2 up-regulates expression of myocardin and SMC contractile markers and inhibits VSMC proliferation and migration, we next sought to determine whether the effect of SMYD2 on VSMC behavior is myocardin dependent. Therefore, we downregulated the expression of myocardin with *myocardin* siRNA (*siMyocd*) in WT (vector control) and *Smyd2* overexpressing VSMCs. RT-qPCR analysis confirmed the knockdown of *myocardin* mRNA by *siMyocd* in both vector control and *Smyd2* overexpressing VSMCs, compared with control siRNA (*siCon*) (Figure 5A). *Smyd2* overexpression significantly increased the expression levels of *Acta2* and *Myh11* mRNA in VSMCs, but these effects were abrogated by *myocardin* knockdown (Figure 5B, C).

Furthermore, CCK8 proliferation and Transwell migration assays showed that VSMC proliferation and migration were significantly attenuated by *Smyd2* overexpression, but these effects were reversed by *myocardin* knockdown (Figure 5D-F). Taken together, these results suggest that SMYD2 induces VSMC marker gene expression and inhibits VSMC proliferation and migration in a myocardin-dependent manner.

### Myocardin interacts with SMYD2 and enhances SMYD2 binding to CArG regions of VSMC marker gene promoters

**Figure 6.**
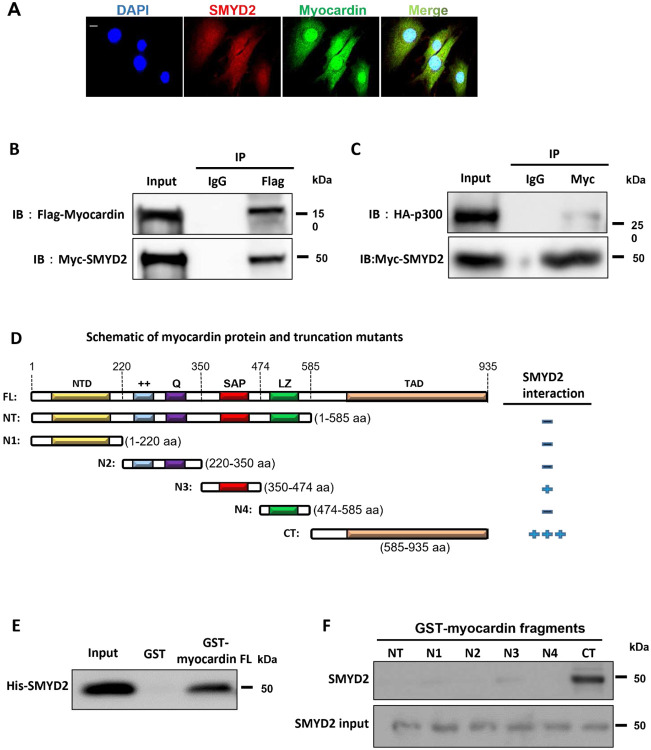

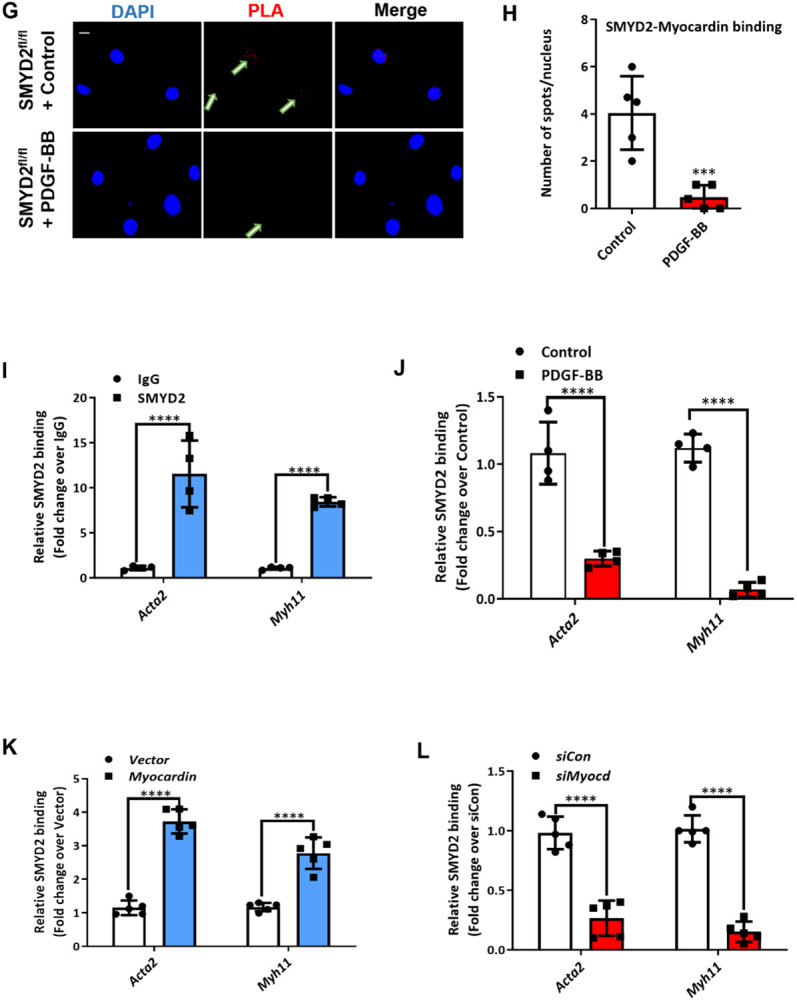
SMYD2 physically interacted with myocardin and enhanceed its binding to CArG regions of VSMC marker promoters **A,** Representative immunofluorescence staining for SMYD2 (red) and myocardin (green) in mouse primary aortic VSMCs. The nuclei were counterstained with DAPI (blue). Scale bar, 100 μm. **B - C**, 10T1/2 cells were co-transfected with Myc-SMYD2 and Flag-myocardin (**B**), or co-transfected with Myc-SMYD2, Flag-myocardin, and HA-p300 (**C**). Co-IP was performed using normal rabbit IgG as the control or anti-Flag antibody (**B**) or anti-Myc antibody (**C**). Following IP, the immune complex was detected using antibodies against SMYD2 and myocardin (**B**) or SMYD2 and HA (for p300) (**C**). **D,** Schematic diagram of mouse full length (FL) myocardin and truncation mutants used for GST pull-down assay with SMYD2. NTD, N-terminal domain; ++, basic domain; Q, polyQ domain; SAP, SAF-A/B, Acinus, and PIAS domain; LZ, leucine zipper domain; TAD, transcriptional activation domain; NT, N-terminus; CT, C-terminus; N1-4, N-terminal mutants 1–4. **E,** Purified GST or GST-myocardin FL (full-length), immobilized on glutathione beads, was incubated with purified His-SMYD2 in GST pull-down assay. Bound SMYD2 was pulled down and subjected to immunoblotting with anti-SMYD2 antibody. **F,** GST pull-down assay using truncation mutants of NT myocardin (N1-4), NT, and CT-myocardin GST fusion proteins, incubated with purified His-SMYD2. Bound SMYD2 was pulled down with glutathione beads and immunoblotted with anti-SMYD2 antibody. **G,** VSMCs were serum starved overnight and then treated with vehicle control or PDGF-BB (10 ng/ml) for 24 h. PLA assays were performed to detect SMYD2 and myocardin binding in intact cells. Arrows denoted representative positive spots (red) indicating SMYD2-myocardin interaction in vivo. Scale bar, 100 μm. **H,** Quantification of positive red spots per nucleus in PLA assays (**G**) (n=5 independent experiments). **I,** Chromatin immunoprecipitation (ChIP) assay of SMYD2 binding to CArG regions of *Acta2* and *Myh11* promoters in VSMCs. Cross-linked chromatin was harvested for immunoprecipitation with SMYD2 antibody or IgG control. The precipitated DNA was amplified by real-time PCR using *Acta2* and *Myh11* promoter specific primers that span the CArG regions. SMYD2 binding was presented relative to control IgG (set to 1) (n=4 independent experiments). **J,** Primary murine VSMCs were serum starved for 24 h followed by treatment with vehicle control or PDGF-BB (10 ng/ml) for 24 h. Subsequently, cells were harvested for ChIP assay to examine SMYD2 binding to CArG regions of *Acta2* and *Myh11* promoters (n=4 independent experiments). **K,** VSMCs were transduced with *GFP* vector adenovirus or *GFP-myocardin* adenovirus. Post-transduction 48 h, ChIP assay of SMYD2 binding to CArG regions was performed as described in (**I**) (n=5 independent experiments). **L,** VSMCs were transfected with control siRNA (*siCon*) or *myocardin* siRNA (*siMyocd*) for 48 h, followed by ChIP assay of SMYD2 binding to CArG regions as described in (**I**). *P<0.05 vs. siCon group (n=5 independent experiments). For all bar graphs, data are expressed as the means ± SEM, ***P < 0.001 and ****P < 0.0001 (unpaired, two-tailed Student’s t-test).

To further explore the relationship between SMYD2 and myocardin, we performed immunofluorescence staining to examine their subcellular location in VSMCs. As shown in Figure 6A, SMYD2 was co-localized with myocardin in both the nucleus and cytoplasm of VSMCs. We next examined whether SMYD2 binds to myocardin to promote its transactivation of SMC marker genes. Co-immunoprecipitation (co-IP) assay showed that SMYD2 co-precipitated with myocardin in multipotential 10T1/2 cells transfected with Myc-SMYD2 and Flag-myocardin (Figure 6B). Myocardin has been reported to bind directly with p300, a transcriptional coactivator and histone acetyltransferase, via transcription activation domain (TAD) to promote transactivation of SMC genes.^63^ We tested whether SMYD2 binds to p300, either directly or indirectly via myocardin. co-IP assay showed that p300 was immunoprecipitated with SMYD2 (Figure 6C). We could not detect any interaction between SMYD2 and SRF through co-IP in either 10T1/2 overexpressing tagged SMYD2 and SRF, or in cultured VSMCs with endogenous proteins. Using bacterially purified proteins and protein domain mutants in a GST pull-down assay as we reported recently,^43^ we detected a direct binding between SMYD2 and myocardin (Figure 6D and E) mediated primarily via C-terminal (CT) domain of myocardin containing TAD (Figure 6F). The results from co-IP and GST pull-down assays (Figure 6B-F) were obtained using cell homogenates. To determine if SMYD2 interacts with myocardin within intact cells, we performed in situ proximity ligation assay (PLA) which permits detection of proteins located within about 40 nm in cells.^54^ We observed an interaction between SMYD2 and myocardin in primary VSMCs by PLA method, which was significantly attenuated upon treatment with PDGF-BB (Figure 6G and H).

Given that SMYD2 interacts physically with myocardin (Figure 6B-H) and promotes SMC marker gene expression (Figure 4D-H), we hypothesize that SMYD2 may also be recruited to CArG regions of VSMC marker gene promoters, probably mediated by myocardin. Quantitative chromatin immunoprecipitation (ChIP) assays revealed SMYD2 enrichment in the CArG regions of *Acta2* and *Myh11* gene promoters (Figure 6I), which was significantly reduced by PDGF-BB treatment (Figure 6J). Moreover, myocardin overexpression in VSMCs significantly increased SMYD2 binding to the CArG box of VSMC marker gene promoters (Figure 6K), whereas myocardin knockdown drastically decreased SMYD2 binding to these promoters (Figure 6L). Of note, SMYD2 expression was not affected by myocardin overexpression or knockdown. Collectively, these results demonstrate that myocardin physically interacts with SMYD2 and enhances recruitment of SMYD2 in CArG regions of SMC contractile gene promoters.

### SMYD2 maintains an active epigenetic state of chromatin within VSMC marker gene promoters

**Figure 7.**
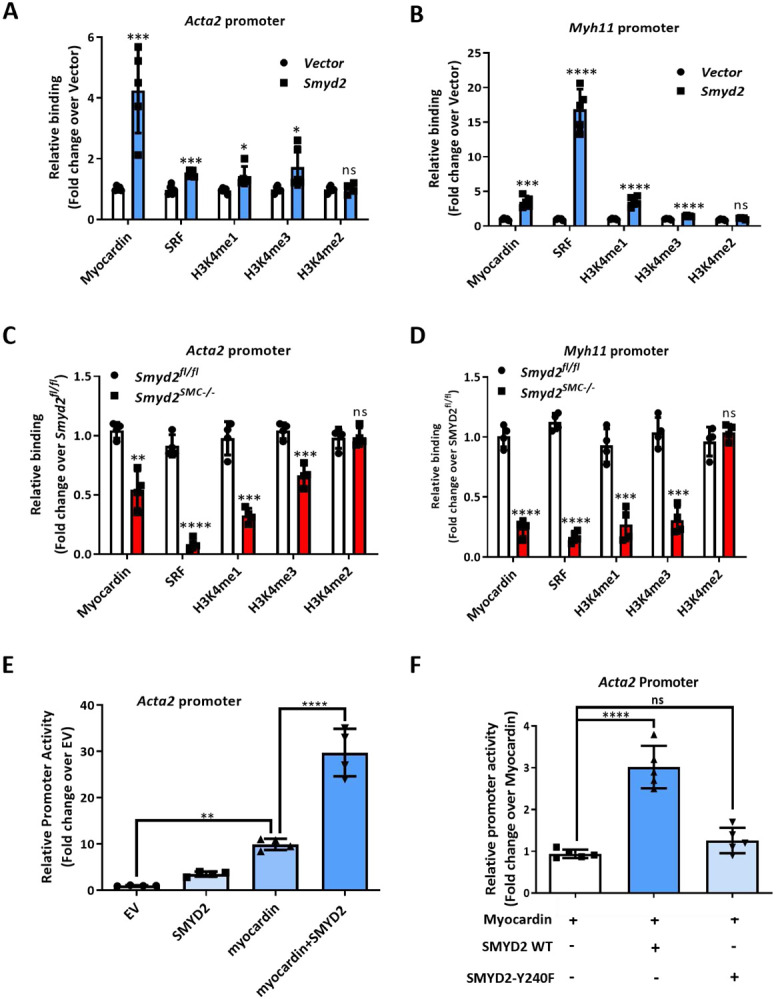
SMYD2 maintained an active epigenetic state of chromatin within VSMC marker gene promoters **A** and **B,** ChIP assay of the enrichment of myocardin, SRF, H3K4me1, H3K4me2 and H3K4me3 at CArG regions of *Acta2* gene promoter (**A**) and *Myh11* gene promoter (**B**) in VSMCs. Lentivirus encoding *GFP-Smyd2* or *GFP* control were transduced into mouse VSMCs. Post-transduction 48 h, cells were serum starved overnight, followed by ChIP assay (n=5 independent experiments). **C** and **D,** Primary murine VSMCs isolated from *Smyd2*^*fl/fl*^ or *Smyd2*^*SMC−/−*^ mice were serum starved overnight and subjected to ChIP assay for the enrichment of myocardin/SRF and H3K4me1/2/3 at CArG regions of *Acta2* (**C**) and *Myh11* (**D**) promoters (n=4 independent experiments). **E,** The luciferase reporter construct harboring *Acta2* gene promoter (containing CArG region) was transfected into 10T1/2 cells with or without SMYD2 and/or myocardin expression plasmids. Post-transfection 48 h, dual luciferase reporter assays were performed to determine *Acta2* gene promoter activity. The promoter activity of the reporter at baseline without SMYD2 or myocardin transfection was set to 1 (EV, set to 1, n=4 independent experiments). **F,** The *Acta2* CArG gene promoter-luciferase reporter was co-transfected with myocardin and/or either wild-type (WT) or enzymatically inactive mutant SMYD2 (Y240F) expression plasmids into 10T1/2 cells. Post-transfection 48 h, cells were harvested for dual luciferase assays. Reporter activity was normalized to a renilla luciferase internal control and expressed relative to the transfection with myocardin alone (set to 1). *P<0.05 vs. myocardin alone (set to 1) (n=5 independent experiments). For all bar graphs, data are expressed as the means ± SEM, ns=no significance, *P<0.05, **P<0.01, ***P < 0.001 and ****P < 0.0001 (unpaired, two-tailed Student’s t-test for A-D; one-way ANOVA with Tukey’s post hoc test for E and F).

Previous studies reported that active epigenetic marks H3K4me1 (histone 3 lysine 4 mono-methylation) and H3K4me3 (histone 3 lysine 4 tri-methylation) at the promoters of VSMC markers initiate an open state of chromatin and promote transcription of these markers.^8^ However, the identity of the histone methyltransferases involved in this process remains unclear. Since SMYD2 is known to mediate H3K4 methylation,^21^ and we demonstrated prominent enrichment of SMYD2 in CArG regions of VSMC marker gene promoters, we speculated that SMYD2 may play a pivotal role in opening chromatin (maintaining an euchromatin) around VSMC marker gene promoters by depositing epigenetic marks H3K4me1/3 in CArG regions. We therefore examined SMYD2-regulated epigenetic changes at SMC gene promoters using ChIP assays. ChIP/qPCR analysis demonstrated that SMYD2 overexpression significantly increased the enrichment of myocardin, SRF, and active epigenetic marks H3K4me1/3 (mono- and tri-methylation) in CArG regions of *Acta2* and *Myh11* gene promoters in VSMCs (Figure 7A, B). However, we did not observe significant changes in the enrichment of other SMYD2-mediated marks (H3K36 and H4K20) in CArG regions. Conversely, the enrichment of myocardin/SRF and H3K4me1/3 at CArG regions of *Acta2* and *Myh11* promoters was significantly decreased in *Smyd2*^*SMC−/−*^ VSMCs compared to *Smyd2^fl/fl^* VSMCs (Figure 7C, D). These results suggest that the activation of endogenous SMC marker genes by myocardin is accompanied by SMYD2-mediated methylation of histones at the corresponding gene promoters, specifically around CArG regions.

To confirm that the function of SMYD2 in regulating VSMC contractile phenotype is dependent on methyltransferase activity, VSMCs were pre-treated with SMYD2 selective methyltransferase inhibitor Bay-598.^45^ We found that SMYD2 inhibitor significantly attenuated VSMC marker SMAα expression (Supplementary Figure S7) and promoted proliferation and migration of VSMCs *in vitro* (Supplementary Figure S4). Moreover, luciferase reporter assay demonstrated that co-transfection of SMYD2 and myocardin synergistically transactivated *Acta2* promoter compared with myocardin alone (Figure 7E). However, an enzymatically inactive SMYD2 (Y240F) ^34^ failed to achieve synergistic effect on promoting *Acta2* promoter activity when it was co-transfected with myocardin into 10T1/2 cells (Figure 7F), indicating that the ability of SMYD2 to enhance myocardin transactivation is dependent on its methyltransferase activity.

**Figure 8.**
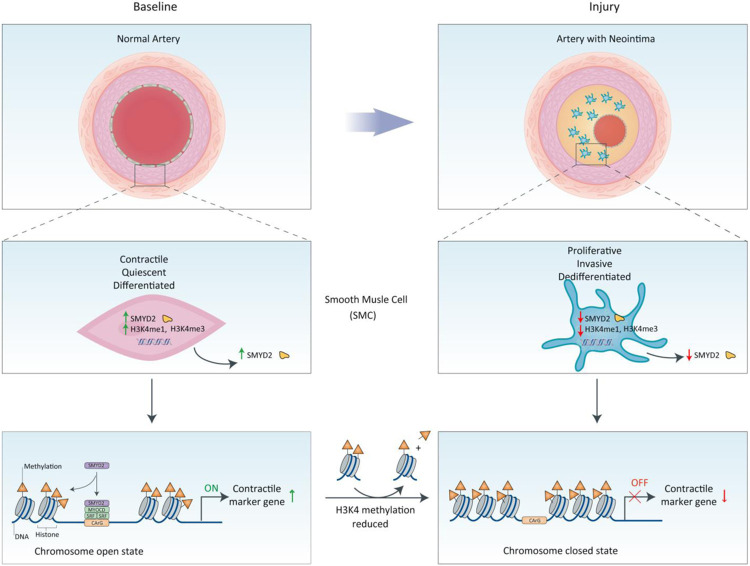
Schematic diagram depicting the role of SMYD2-myocardin regulatory network in VSMC differentiation and phenotypic switching. Under physiological condition, SMYD2 up-regulates myocardin expression and methylates H3K4 (mono- and trimethylation) to maintain an active state of chromatin, which facilitates the binding of myocardin and SRF at CArG regions of VSMC marker genes and promotes transactivation of myocardin and marker gene expression, thereby inhibiting proliferation and migration of VSMC and neointima formation. However, in response to injury or PDGF-BB, down-regulation of SMYD2 inhibits myocardin expression and reduces active epigenetic marks (H3K4me1/3) to initiate a closed state of chromatin at CArG regions, resulting in SM marker gene suppression, VSMC phenotypic switching, and neointima formation.

Taken together, our results demonstrate that SMYD2 is expressed in contractile VSMCs. Under this circumstance, SMYD2 upregulates myocardin expression and methylates H3K4 (H3K4me1 and H3K4me3) to maintain an active state of chromatin (Figure 8). This facilitates the binding of myocardin to SRF at the promoters of VSMC marker genes to promote SM marker gene transcription and inhibit the proliferation and migration of VSMCs and neointima formation. Meanwhile, myocardin facilitates SMYD2 recruitment to VSMC marker gene promoters via direct interaction. In contrast, in response to arterial injury or growth factors such as PDGF-BB, downregulation of SMYD2 attenuates myocardin expression and reduces deposition of active epigenetic markers (H3K4me1/3) to induce a closed state of chromatin at the CArG regions of SM marker gene promoters. This leads to the inhibition of VSMC marker expression and VSMC phenotypic switch, resulting in enhanced proliferation and migration and exaggerated neointima formation.

## Discussion

Previous studies have demonstrated that SMYD2, a lysine methyltransferases, ^64^ methylates histones and non-histone proteins and contributes to the progression of a variety of disease conditions such as cancers,^25,27,30^ inflammation,^34^ HIV-1 latency,^22^ and polycystic kidney disease.^29^ It was recently reported that SMYD2 was downregulated in abdominal aortic aneurysm and may be a protective factor in this disease condition.^35,36^ However, the role of SMYD2 in vascular disease is unclear. Our present study provides the evidence on a pivotal role of SMYD2 in vascular diseases and remodeling, especially neointima formation. Our results revealed that SMYD2 expression was decreased in injured mouse carotid arteries, consistent with previous reports that SMYD2 was downregulated in vascular disease.^36^ By utilizing SMC-specific Smyd2 KO mice and Smyd2 overexpression lentivirus, we demonstrated that SMC-specific Smyd2 ablation exaggerated injury-induced neointima formation in carotid arteries, whereas Smyd2 overexpression attenuated vascular intimal hyperplasia. We further examined whether SMYD2 regulates VSMC proliferation and migration, the two key cellular events that accompany neointima formation and atherosclerosis. ^41^ Our results showed that Smyd2 deletion in VSMCs led to significant increase in cell proliferation and migration at baseline and upon PDGF-BB treatment. Moreover, Smyd2 overexpression markedly inhibited VSMC proliferation and migration induced by PDGF-BB. Previous studies have demonstrated that SMYD2 is involved in Rb or p53-dependent cell-cycle control in cancer cells via transcriptional regulation.^23,25^ To reveal the mechanisms by which SMYD2 regulates VSMC phenotype/differentiation, proliferation, and migration, we performed whole transcriptome RNA sequencing. Our results indicated that Smyd2 deletion significantly inhibited the expression of myocardin and VSMC contractile genes. Using loss- and gain-of-function approaches, we further validated that SMYD2 promoted the expression of VSMC marker genes such as *Acta2*, *Myh11,* and *Tagln.*

Myocardin is a potent transcriptional coactivator expressed specifically in cardiac and smooth muscle cells, and functions primarily through direct contact with the ubiquitously expressed transcription factor SRF over CArG boxes within a number of genes encoding for contractile and cytoskeletal proteins to regulate VSMC phenotypes.^4^ Myocardin also negatively regulates VSMC proliferation and migration as well as inflammatory response.^65,66^ In this study, for the first time we demonstrated that SMYD2 upregulated myocardin expression in VSMCs, and SMYD2-mediated inhibition of VSMC phenotypic switch, proliferation and migration was myocardin dependent. We also observed direct interaction between SMYD2 and myocardin. A previous study reported that the expression of myocardin was markedly downregulated in Nkx2.5-null mouse hearts.^67^ Interestingly, we also observed a decrease of Nkx2.5, an evolutionarily conserved cardiac transcription factor, in Smyd2 deficient VSMCs based on RNA sequencing data. These results may partially explain how SMYD2 regulates myocardin expression, which needs further investigation in the future.

High level of H3K4 methylation, especially trimethylation (H3K4me3), is associated with the 5’ region of nearly all active genes, and is critical for maintaining an open chromatin state for activation of gene transcription.^68^ It has been reported that a ubiquitously expressed histone methyltransferase adaptor protein WDR5 regulates H3K4 methylation states in VSMC marker gene promoters.^8^ We recently demonstrated that knockdown of WDR5 or sequester of WDR5 by lncRNA NEAT1, which downregulates H3K4me levels as reported before,^8,15^ decreases histone acetylation ^69^ and initiates an epigenetic off state.^15^ However, it remains to be elucidated which specific methyltransferase contributes to H3K4 methylation in VSMCs. Our present study showed that myocardin physically interacted with SMYD2 and enhanced its binding to CArG regions of VSMC marker gene promoters. We also observed that SMYD2 increased the levels of H3K4me1 and H3K4me3, but not H3K4me2, in CArG regions of VSMC marker gene promoters, and further enhanced myocardin and SRF enrichment in the CArG regions. Previous study showed that H3K4me2 remained unchanged during VSMC phenotypic switch and may serve as a platform for H3K4me1 and H3K4me3, ^8^ consistent with our findings. Myocardin increases histone acetylation of SMC-specific promoters through a direct interaction with histone acetyltransferase p300.^63,70^ Intriguingly, our current study also revealed that p300 co-precipitated with SMYD2 (probably through myocardin). These findings suggest that myocardin, via direct interaction with both SMYD2 and p300, may regulate chromatin structure and SMC marker gene expression.^6^ Our data suggest a positive feed-back loop that SMYD2 upregulates myocardin expression and myocardin increases SMYD2 enrichment in the promoter regions of VSMC markers, which help maintain an open state of chromatin and further promote the binding of myocardin/SRF to the gene loci. Through these cooperative effects, SMYD2 attenuates VSMC phenotypic switch and inhibits neointima formation after vascular injury. It has been reported that SMYD2 inhibits inflammation, an essential process in restenosis and aneurysm, by suppressing the production of inflammatory factors IL-6 and TNF-alpha.^34^ These findings also support the protective role of SMYD2 in restenosis after vascular injury that was observed in our study.

However, some limitations exist in the present study. First, Tagln-Cre was used to target Smyd2 in VSMCs of C57BL6 mice. Accumulating studies have reported Tagln expression in other cell types such as cardiomyocytes, adipocytes, keratinocytes, and myeloid cells. ^71,72^ However, a previous study targeting Smyd2 in cardiomyocytes showed that cardiac deletion of Smyd2 is dispensable for mouse heart development. ^32^ Nevertheless, a more SMC-specific Cre line (such as Myh11-CreER^T2^) or vascular SMC-specific Cre line (most recently reported Itga8-CreER^T2^) ^73^ could be used to confirm our findings in future studies. Second, we used lentivirus to overexpress SMYD2 in carotid vessels of mice with lack of cell specificity. A mouse line with VSMC-specific Smyd2 knock-in might be a better tool to explore the protective role of SMYD2 in neointima formation. Third, our study focused on SMYD2 and myocardin interaction in neointima formation; however, as SMYD2 may potentially bind to other proteins in different cell types under different contexts, whether there are other mechanisms of SMYD2 in regulating neointima formation needs to be further explored. Most recently, another member of SMYD family, SMYD3, has been shown to promote VSMC proliferation and migration during injury-induced vascular remodeling, via directly upregulating cell proliferation and migration - related genes. ^74^ However, in our RNA-seq study, we did not observe any significant changes in mRNA levels of other SMYD family members in primary VSMCs isolated from *Smyd2*^*SMC−/−*^ mice compared with *Smyd2*^*fl/fl*^ control mice.

In summary, our present work revealed a previously unrecognized role of SMYD2 in regulating SMC phenotype and vascular homeostasis, and the findings broaden the knowledge of tissue distribution of SMYD2 and molecular mechanism of action of SMYD2 in vascular diseases. SMYD2 regulates VSMC phenotypic switch and neointima formation via interaction with myocardin. Moreover, local delivery of SMYD2 lentivirus particles into the injured arteries attenuated intimal hyperplasia, suggesting the possibility of using site-specific delivery of SMYD2 via drug–coated stents or balloon to prevent restenosis after angioplasty. Our current study provides a new therapeutic target for diseases associated with abnormal VSMC phenotype and proliferation, such as post-angioplasty restenosis, aneurysm, and hypertension.

## Data Availability

All data associated with this study are present in the paper or the Supplementary Materials. RNA-seq data are accessible at the GEO under accession number: GSE161004. Any additional information for this study is available by contacting the corresponding authors upon reasonable request.

## Abbreviations:

CArG: CC(A/T)_6_GG
ChIP: Quantitative chromatin immunoprecipitation
ChIP-qPCR: Chromatin immunoprecipitation-quantitative PCR
CT: C-terminal
H3K36: Histone 3 at lysine 36
H3K4: Histone 3 lysine 4
H4K20: Histone 4 at lysine 20
HASMC: Human aortic smooth muscle cell
LCA: Left common carotid artery
MRTF-A/B: Myocardin-related transcription factor A/B
PDGF-BB: Platelet-derived growth factor BB
PLA: Proximity ligation assay
RCA: Right common carotid artery
SMYD2: SET and MYND domain-containing protein 2
Smyd2^SMC−/−^: SMC-specific Smyd2-deficient mice
Smyd2^fl/fl^: Smyd2 homozygous floxed mice
SRF: Serum response factor
TAD: Transcription activation domain
VSMC: Vascular smooth muscle cell
